# Impact of exercise training on symptoms of depression, physical activity level and social participation in people living with HIV/AIDS: a systematic review and meta-analysis

**DOI:** 10.1186/s12879-022-07145-4

**Published:** 2022-05-16

**Authors:** Sam Chidi Ibeneme, Victor Chukwuebuka Uwakwe, Hellen Myezwa, Franklin Onyedinma Irem, Fortune Elochukwu Ezenwankwo, Tunde Adedayo Ajidahun, Amarachi Destiny Ezuma, Uchenna Prosper Okonkwo, Gerhard Fortwengel

**Affiliations:** 1 Department of Medical Rehabilitation, Faculty of Health Sciences, University of Nigeria, Enugu Campus, 1 College Road, New Layout, Enugu, 400001 Nigeria; 2 Department of Physiotherapy, Faculty of Health Sciences, School of Therapeutic Studies, University of the Witwatersrand, 7 York Road, Parktown, Johannesburg, 2193 Gauteng South Africa; 3 Department of Physiotherapy, University of Nigeria Teaching Hospital, KM 35 Enugu Port-Harcourt Expressway, Ituku/Ozalla, 400001 Enugu Nigeria; 4 Division of Exercise Science and Sports Medicine, University of Cape Town/Sports Science, Institute of South Africa, Boundary Road, Newlands, Cape Town, 7725 Western Cape South Africa; 5 Department of Medical Rehabilitation, Faculty of Health Sciences, Nnamdi Azikiwe University, Okofia, Nnewi Campus, Nnewi, 435101 Anambra State Nigeria; 6 University of Nigeria, University of Nigeria Centre for Clinical Trials (UNNCET), 1 College Road, New Layout, Enugu, 400001 Nigeria; 7 Fakultat III, Hochschule Hannover - University of Applied Sciences and Arts, Expo Plaza, Hannover, 30539 Lower Saxony Germany; 8 Department of Physiotherapy, Faculty of Health Sciences & Technology, King David University of Medical Sciences, Amasir i- Afikpo Road, Uburu, 491101 Ebonyi State Nigeria

**Keywords:** Exercise training, Mental health, Physical activity level, Social participation, HIV/AIDS

## Abstract

**Background:**

Symptoms of depression are prevalent in people living with human immune deficiency virus/acquired immune deficiency syndrome (PLWHA), and worsened by lack of physical activity/exercises, leading to restriction in social participation/functioning. This raises the question: what is the extent to which physical exercise training affected, symptoms of depression, physical activity level (PAL) and social participation in PLWHA compared to other forms of intervention, usual care, or no treatment controls?

**Method:**

Eight databases were searched up to July 2020, according to the Preferred Reporting Items for Systematic Review and Meta-Analysis (PRISMA) protocol. Only randomised controlled trials involving adults who were either on HAART/HAART-naïve and reported in the English language, were included. Two independent reviewers determined the eligibility of the studies, extracted data, assessed their quality, and risk of bias using the Physiotherapy Evidence Database (PEDro) tool. Standardised mean difference (SMD) was used as summary statistics for the mean primary outcome (symptoms of depression) and secondary outcomes (PAL and social participation) since different measuring tools/units were used across the included studies. Summary estimates of effects were determined using a random-effects model (I^2^).

**Results:**

Thirteen studies met the inclusion criteria with 779 participants (n = 596 participants at study completion) randomised into the study groups, comprising 378 males, 310 females and 91 participants with undisclosed gender, and with an age range of 18–86 years. Across the studies, aerobic or aerobic plus resistance exercises were performed 2–3 times/week, at 40–60 min/session, and for between 6-24 weeks, and the risk of bias vary from high to low. Comparing the intervention to control groups showed significant difference in the symptoms of depression (SMD = − 0.74, 95% confidence interval (CI) − 1.01, − 0.48, p ≤ 0.0002; I^2^ = 47%; 5 studies; 205 participants) unlike PAL (SMD = 0.98, 95% CI − 0.25, 2.17, p = 0.11; I^2^ = 82%; 2 studies; 62 participants) and social participation (SMD = 0.04, 95% CI − 0.65, 0.73, p = 0.91; I^2^ = 90%; 6 studies; 373 participants).

**Conclusion:**

Physical exercise training could have an antidepressant-like effect in PLWHA but did not affect PAL and social participation. However, the high heterogeneity in the included studies, implies that adequately powered randomised controlled trials with clinical/methodological similarity are required in future studies.

***Trail Registration number*:**

INPLASY202040048.

**Supplementary Information:**

The online version contains supplementary material available at 10.1186/s12879-022-07145-4.

## Background

People living with the human immune deficiency virus/acquired immune deficiency syndrome (HIV/AIDS) (PLWHA) present with symptoms of psychological and physiological dysfunction [1, 2]. The common physical symptoms of HIV include headache, pain, fatigue, nausea, diarrhoea, rash, lipodystrophy, and lipoatrophy [1, 2]. These symptoms allude to multi-systemic alterations of the underlying biological processes due to either HIV, antiretroviral therapy or both [1, 2]. The subsequent disabilities which arise in PLWHA, involve physical, cognitive, mental and emotional signs [3]. These disabilities negatively impact their coping strategies [3] with adverse implications for social interactions [4] and are worsened by lack of physical exercises and restriction in social participation [5], leading to multi-system impairments [6]. Such impairments entail a decline in the physical activity level (PAL), thereby reinforcing the vicious cycle that advances the symptoms and disabilities associated with HIV. Therefore, exercise training, which may improve PAL, could address some social, mental and health-related challenges in PLWHA.

Physical activity indicates any human movement generated by skeletal muscle contraction at an increased metabolic cost, over a basal value at rest [5]. Invariably, exercise is considered a subcategory of physical activity and a constituent of most activities of daily living (ADL), which is goal-directed and individual-specific. As a result, Caspersen, Powell [7] defined physical exercise as organized, structured, and repetitive physical activity, and having a goal to improve or maintain physical fitness. The U.S. Department of Health and Human Services [8] strongly recommends that adults (18–64 years of age) engage in 150 min of moderate-intensity aerobic physical activity each week or 75 min of vigorous-intensity aerobic physical activity, or a combination of the two. For the same reasons, 10,000 steps per day is a recommended target for improving health outcomes and physical fitness in healthy individuals [9, 10]. Exercises improve the PAL [11–13] and physical activity score in PLWHA [14]. It tunes up the musculature in boosting functional capacity and mobility, which is often restricted by the effects of HIV, HAART and associated complications [15]. Importantly, exercises enhance recovery from disability; optimize mental, physical, economic or social outcomes for PLWHA [16].

Social participation relates to an individual’s ability to fulfil personal goals and socially-defined roles in a community. A person's participation in real-life situations is determined by health conditions, body structure, functions, activities, and contextual environmental factors [17] stated in the International Classification of Functioning, Disability and Health (ICF). In their study [18], Van As and Myezwa reported that social participation in PLWHA was restricted because of impairments, activity limitations, and participation restrictions arising from pain, weakness of the muscles and fear of aggravating an existing pain when engaging in movement-driven activities. Functional limitations, in PLWHA, influence care needs and capacity to perform social roles (such as engaging in a job) [19]. Additionally, Myezwa et al. [20] observed that insufficient energy to participate in ADL or continue being socially active while coping with the disease is a recurring concern in PLWHA. Impairment and consequent disability arise from the negative effects of HIV [21], and adverse drug interactions from polypharmacy, leading to fatigue/exhaustion [22, 23]. Nevertheless, fatigue/low energy is amenable to physical exercises [24], which improve vitality, social participation [11–13], and minimise social dysfunction [25–27]. Though, exercises were shown to have no effect on social participation and social contact [28, 29]. However, considering that physical exercises relieve pain which is linked with social functioning [30, 31], and pain negatively impacts mental health [32], then exercises should have a bidirectional relationship with both pain and mental health.

Mental health is not only the absence of psychopathology but also relates to the emotive, psychological and social well-being of an individual/group [33–35], as the factors that negatively affect them will impair mental health. This makes it easier to understand why neuropsychiatric disorders in PLWHA is related to lipodystrophy and HAART [36, 37]. Severe weight loss, and unhealthy physical appearance in PLWHA are attributed to lipodystrophy and lipoatrophy arising from adipose tissue alterations, which has a negative influence on self-image, self-esteem, and social functioning [38, 39]. The sequelae involve poor mental health, lower functional wellbeing, and lack of self-confidence, which limit ADL and social participation [40, 41]. This may eventually lead to social withdrawal/isolation, which in a sense informs why a higher prevalence of depression/mental disorders occur in PLWHA [42–44]. However, exercise ameliorates the symptoms of severe depression in PLWHA [45]. An epidemiological study showed that regular physical activity and depressive disorders are negatively related in adults [46]. Despite claims about the effects of physical exercises in PLWHA; it is unclear how much physical activity they engage in [15] or may require to ameliorate symptoms of depression, improve PAL, and social participation. Consequently, this review aimed to assess whether physical exercise training has any impact on symptoms of depression, social participation and PAL in PLWHA. The review question is—What is the extent to which physical exercise training affected symptoms of depression, PAL and social participation in PLWHA compared to other forms of interventions, usual care, or no treatment controls?

## Methods

The study was conducted using the Preferred Reporting Items for Systematic Reviews and Meta-Analyses (PRISMA) protocol. The protocol for this systematic review was registered on the international platform for registering systematic reviews and meta-analyses (INEPLASY register) on 9 April 2020 (registration number: INPLASY202040048).

### Eligibility criteria

The following eligibility criteria were used to select the studies for the review:

#### Inclusion criteria

##### Type of studies

The review was restricted to original studies published in peer-review journals and conference proceedings in English. This review included only randomised controlled trials (RCTs) studies that evaluated the effects of exercise training on: depression, PAL and social participation/social functioning in PLWHA.

##### Types of participants

PLWHA, > 18 years and are either on HAART or HAART-naïve. In as much as a specific limitation on the location of the studies was not considered, nevertheless, the included studies were mainly based in clinics/hospitals, home or community care settings.

##### Types of intervention

RCTs involving physical exercise intervention among PLWHA were included in the review but was not limited to any exact dosage, type, intensity, frequency and length of intervention or follow-up period post-intervention. The exercise intervention may be clinic/hospital-based, community-based or home-based, and the exercise type may be aerobic, resistance exercise or a combination of both. Similarly, RCTs of resistance exercise intervention were not restricted to either isometric and isotonic muscle strengthening exercises or weight training in PLWHA.

##### Types of control

This review included studies that compared the effects of physical exercise training on symptoms of depression, PAL and social participation/social functioning to a control group receiving other forms of interventions, usual care, or no treatment.

##### Type of outcomes

*Primary outcomes* Symptoms of depression*:* included a recurrent sense of unhappiness and dejection and lack of interest which affects how an individual feels or thinks and behaves, leading to many physical and emotional problems. Several psychiatric rating scales are used for measuring this outcome namely—mood state questionnaire—POMS-30, General Health Questionnaire-28—GHQ-28, Beck’s Depression Inventory-BDI, and Centre for Epidemiologic Studies Depression Scale (CES-D).

Secondary outcomes

(i) *Physical activity level* This is operationalised as the quantity of an individual’s day-to-day physical movement. It is used to gain a sense of the amount of energy expended (in terms of intensity and duration) in physical movements employed in the activities of daily living. It is measured using monitor-based devices or wearable technologies such as pedometers, accelerometers, or Global Positioning System (GPS) units, and expressed using heart rate, pedometer step count per day, meters per second squared (m/s^2^) or in G-forces (g), respectively.
(ii) *Social participation/Social functioning* Social participation is defined as the frequency of an individual’s engagement in activities that afford interaction with other community dwellers within and outside the home. Social participation is also taken as social functioning which defines an individual's interactions with their environment and the ability to fulfil their social roles within such environments and responsibilities as work, social activities, and relationships with spouse/partners and family. This outcome is measured using functional performance-based instruments (using subscales within generalized scales) that include six types of activities: hobby, friendship, clubs, volunteer activities, community events and communication with family members and friends. The measuring instruments include the 36-Item Short Form Survey (SF-36), The Medical Outcomes Study HIV Health Survey (MOS-HIV), Duke Activity Status Index [DASI], and The World Health Organization's Quality of Life HIV instrument-Brief (WHOQOL-HIV-BREF.

Studies were included regardless of whether an outcome of interest is the primary or secondary outcome in the include studies, so far as a clear analysis was carried out for each outcome. All outcome variables were aggregated as they were documented in individual studies, and the original form of the data in those individual studies was not altered. Clinical results, detailed by individual studies were analysed and graded.

#### Exclusion criteria

1. Studies that did not evaluate an exercise or physical activity intervention component.
2. Studies that did not last beyond 4 weeks duration.
3. Narratives review synthesis, systematic reviews, opinion papers, letters and any publication without primary data and/or explicit description of the methods.
4. In considering duplicate publications from the same study, the most recent or most comprehensive publications were used.

### Information sources and search strategy

An extensive search strategy to spot eligible studies was implemented in two phases in line with the PRISMA protocol, including; (i) the search of the bibliographic database and grey literature, and (ii) the choice of studies for inclusion based on eligibility criteria. Searches involved some combinations of search terms from medical subject headings (MeSH) and keywords with a mix of symbolic logic within the title, abstract and text for the population, intervention, control and outcomes, first in a preliminary or pilot search to determine the sensitivity of the search strategy. The search strategy was used differently for the three selected study outcomes. PubMed search strategy is shown in Additional file 1: Appendix I. This strategy was modified to the syntax and subject heading of other databases. Studies were searched in PubMed, Emcare, Cochrane Library, Embase, CINAHL, AMED, PsycINFO and MEDLINE. Additional searches were made of the reference list of identified studies. This procedure was implemented by the recommended guidelines of the Cochrane Handbook for Systematic Reviews [47], and advice for Health Care Review by the Centre for Reviews and Dissemination [48].

### Study record and data management

The search results were exported to the RefWorks™ manager where the Checks for duplication of the identified studies were done. Using the RefWorks™ manager, the bibliographic records were exported to Microsoft Excel 2007 for organization and sorting of articles according to specific eligibility criteria.

### Selection process

**Fig. 1:**
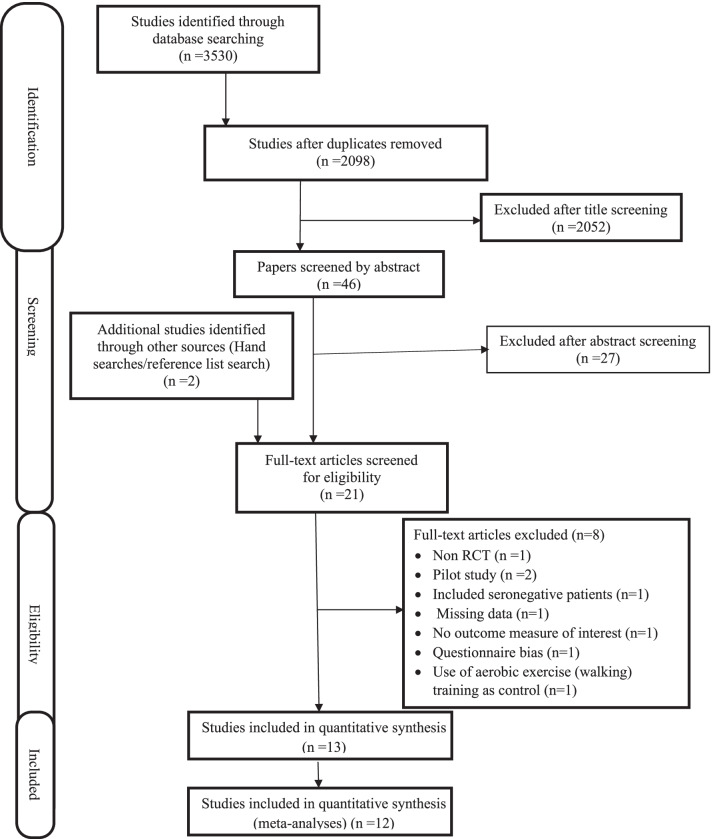
PRISMA diagram for mental health, physical activity level, social participation

The screening was performed in two phases. V.U. (reviewer 1) carried out the first phase of screening based on the title and abstract to spot articles that met the eligibility criteria. The screening results were independently cross-checked by I.F.O (reviewer 2). The two reviewers then read through the full text of the chosen studies for further screening, using the eligibility criteria. In order to reduce assessor bias, differences in opinions about inclusion or exclusion of any identified study were resolved either through discussion and reflection or by consulting with D.I.S (reviewer 3). The reasons why studies were excluded were adequately stated, and a description of the study selection procedure is presented in Fig. 1.

### Data collection processes

(i) *Quality appraisal of included studies* The quality and risk of bias in the included studies were evaluated using the PEDro scale for quality appraisal of clinical trials [49]. The PEDro scale comprises a 10-item checklist, scored either “yes” or “no” on the internal validity and statistical evidence provided in the study. In evaluating the quality of each study, the “yes” in the checklist are tallied up and scored over 10. The scores for the quality of the study are categorised into three, and defined as follows: high quality (6–10), fair/moderate quality (4, 5), and poor quality (≤ 3). When any study is rated as poor-quality, it suggests a possibility that the study has a high risk of bias, while a high-quality study indicates the probability that the study has a low risk of bias. Two reviewers independently adjudged the risk of bias of each of the included studies. Areas of differences were reconciled by discussion and reflection, or in consultation with the third reviewer. The quality appraisal of the quality of the included studies was done after the study selection was done while conducting data extraction and synthesis.
(ii) *Data item* In the included studies, data were collected on the following variables—authors reference, participants’ characteristics (including age range, gender, sample size), study sample size (also groups sample size where available), components of the intervention, the intervention setting, who delivered the intervention, duration of intervention and follow-up (where available), control, attrition rate, outcome(s) assessed, the outcome(s) measuring methods/techniques and summary of results, conclusions and funding sources.

### Data synthesis and assessment of heterogeneity

The collected data were analysed using the review manager (RevMan) to provide an answer to the review question about the impact of physical exercise training on depression, PAL and social participation in PLWHA. Appropriate statistical techniques were applied for each of the study outcomes. The quantitative data synthesis was done by pooling the mean values of the outcomes, standard deviation, and the number of participants in the included studies. These were used to compute the post-intervention standardised mean difference (SMD) for the variables following the standard analysis procedure in the Cochrane meta-analyses. The SMD was determined where different outcome measuring tools/units were used in the included studies. Analysis of continuous variables was done using the weighted mean difference when outcomes were uniform, with a 95% confidence interval. The overall effects of the interventions were determined, evaluated and combined in a proof table. Interpretation of SMD was in accordance with a previous recommendation [50]: small = 0.00–0.39, moderate = 0.40–0.70, and large ≥ 0.70. Alpha was set at p < 0.05.

### Data and sensitivity analysis

Characteristics of the retained studies were arranged by year of publication and presented in a tabular form providing information on authors’ references, sample size, age, setting, data collection format, outcomes, components of the intervention, component of the control, format and provider of the intervention, intervention and follow-up periods, and results. Three meta-analyses to find pooled effect sizes across studies were done, using a random-effects model. Cochrane’s χ^2^ test (10% significance level) and Higgins I^2^ were applied in assessing the heterogeneity of the data, and values of 25%, > 25–75%, and > 75% indicate low, medium and high heterogeneity, as specified in the Cochrane Handbook for Systemic Reviews of Interventions [48]. Examination and documentation of outcomes were made using the primary outcome. Studies that showed clinical or methodological resemblance in terms of design, intervention, and comparator(s) were pooled together for meta-analysis using a random-effects model. Heterogeneous studies were analysed and interpreted using narrative synthesis to determine the relationship and results between and within the studies included in this review. This was done following the recommendation of the Centre for Reviews and Dissemination [47]. A narrative synthesis was done by determining the relationship across the studies. The key concepts in the studies were compiled, matched and differentiated, expressing the studies into one another and adding-up the translations to identify the exact concepts that extend across individual accounts, and which was used to frame an understanding of the effects of physical exercise on depression, PAL and social participation. Sensitivity analysis was done after the primary quantitative synthesis to evaluate the effect that studies with a high risk of bias have on the general outcomes when they are introduced or removed as part of the analysis.

## Result

### Search result

This review included five publications on depression [43, 51–54], seven publications on social participation/functioning [54–60] and two publications on PAL [61, 62]. All the studies included in this review were carried out in the post-HAART era. Altogether, a total of 779 participants was involved across the thirteen studies included in this review.

### Reasons for exclusion

Reasons for the exclusion of eight studies following full-text screening included: a pilot study (n = 2), non-randomized control trials (n = 1), had missing data whose author(s) failed to provide the missing data on request (n = 1), questionnaire bias (n = 1), included seronegative patients (n = 1), used aerobic exercise training as control (n = 1) and did not study any of the outcome measures of interest (n = 1) (Fig. 1).

### Included studies

Table 1 presents the study characteristics of the thirteen publications included in this review, and further details are provided below:

(i) *Depression* Five studies [43, 51–54] reported an exercise intervention's impact on depression. The duration of the interventions ranged from 6 to 16 weeks, the exercise session was from 15 to 60 min, and the sessions per week were from 2 to 3 times per week. All the five included studies [43, 51–54] had no follow-up information. Three studies [43, 52, 54] had a programme of supervised aerobic and resistance exercises. Two studies [51, 53] involved supervised aerobic exercises only. For aerobic and resistance exercises: One study: Dianatinasab et al. [52] had a “behavioural disease counselling and treatment” control group while Oliveira et al. [54] had a “recreational session consisting of stretches, gaming and dancing” for the control group. For aerobic exercises: Neidig et al. [53] had a “usual activity” control, while Aweto et al. [51] had a control group who received “only counselling”.
(ii) *Social participation* Seven studies [54–60] reported the impact of an exercise intervention on social participation/functioning. The duration of the interventions ranged from 8 to 24 weeks, the exercise session was from 10 to 60 min, and the sessions per week were from 1 to 5 times per week. None of the included studies provided any follow-up information. Four studies [52, 57, 60, 62] focused on a supervised aerobic exercise and resistance/strength-building programme. One Study: Ogalha et al. [58] involved supervised aerobic exercise classes and monthly nutritional counselling only. Another study: Maharaj and Chetty [56] involved aerobic exercises administered weekly and a home programme (fast walking, squatting and jogging), which was given twice a week. One study: Baigis et al. [55] involved a home-based workout program using the FM 340 Fitness Master Ski machine at 75–85% maximum heart rate three times a week. For aerobic and resistance/strengthening exercises, one study: Jaggers et al. [43] had a control group that engaged in unsupervised exercises; another study: Dianatinasab et al. [52] had an "exercise-free" control group. For aerobic exercise plus home programme (brisk walking, squatting and jogging): Maharaj and Chetty [56] had a control group that received heat therapy and shortwave diathermy and also, read magazines at home. For supervised gym and monthly dieting counselling: Ogalha et al. [58] involved a control group who mainly engaged in a discussion on nutritional needs and suggestions, and were given orientation on the relevance of the regular physical activity. One study: Baigis et al. [55] had a control group that received usual/routine care weekly.
(iii) *Physical activity level* Only two studies [61, 62] reported the impact of a physical intervention on PAL. One study: McDermott et al. [61] used aerobic exercises alone, involving two supervised sessions and one unsupervised session. The aerobic exercises were delivered three times per week and a "no intervention" control was used. Another study: Roos et al. [62] used pedometer-based walking protocols and an activity diary that included educational materials and documents for self-monitoring.
(iv) Participants of the included studies

**Table 1:** Characteristics of Included Studies

Author, (year) Location of study	Disease stage ART Status Age range Gender Sample size (N) Retention (attrition)	Intervention Group No of participants allocated (No that completed) Adherence rate	Duration of intervention	Control Group No of participants allocated (No that completed)	Outcome Parameter of interest	Measurement tool for outcome	Summary of result
Chung and Lou (2019) Hong Kong	NR On ART 56–84 years Male (16), Female (5) N = 21 95.24% (4.76%)	45 min of supervised combined aerobic & resistance training, each at a moderate intensity of 50–70% MHR, 2 sessions/week for 8 weeks 11 (10) 96.3%	8 weeks	Unsupervised exercise/advised to continue routine daily activities & self-exercise 10 (10)	Social functioning/social participation	The social functioning domain of SF-36 MOS	Self-image & confidence in social life were improved (p = 0.043)
Oliveira et al. (2019) Brazil	NR On ART 18-60 years Male (21), Female (25) N = 46 50% (50%)	15–20 min of supervised combined exercise training (CET): (Aerobic exercise: 15–20 min of moderate-intensity at 50–65% HRR) (Strength training: 15–20 min of 8–15MR of 2–3 sets) 3 sessions/week for 16 weeks n = 25 (14) 87.4%	16 weeks	Recreational session consisting of stretches, gaming & dancing 21 (9)	Depression and Social Functioning/Social Participation	Becks Depression Inventory (BDI) and The social functioning subscale of WHOQOL-HIV	Symptoms of depression were relieved (p < 0.05), unlike the social functioning
Dianatinasab et al. (2018) Iran	NR NR 20–40 years Women N = 40 75% (25%)	Supervised Combinational exercise (Aerobic exercise: 45 min at 40–45% MHR) (Strengthening exercise: 15 min of 3 sets of 8 repetitions at 50–55% RM) 3 sessions/week for 12 weeks Plus VCT’s routine services n = 20 (14) NR	12 weeks	VCT’s routine services n = 20 (16)	Depression	GHQ-28	Symptoms of severe depression were ameliorated (p = 0.008)
McDermott et al. (2016) Ireland	NR NR 18–65 years Male (8), Female (3) NR (2) N = 13 84.6% (15.4%)	31-52 min of 2 supervised & 1 unsupervised (recorded in a diary by participants) aerobic exercise training sessions/week: Circuit training on a treadmill, cycle ergometer, cross-trainer 3 sessions/week at 40–75% HRR for 16 weeks n = 6 (5) 60%	16 weeks	No exercise/adviced to continue with their daily routine n = 7 (6)	Physical Activity level	Actigraph GT3X + Tri Axis Accelerometer	Physical Activity level was largely unchanged
Aweto et al. (2016) Nigeria	Asymptomatic, non-AIDS & symptomatic, non-AIDS HIV patients On HAART 18 years & above Male (15), Female (25) N = 40 84.5% (15.5%)	30 min of supervised aerobic exercise at 50–60% HRR on a cycle ergometer, 3 sessions/week for 6 weeks n = 20 (18) NR	6 weeks	No therapeutic exercises, only had 30 min session of counselling once in 2 weeks n = 20 (15)	Depression	BDI	Symptoms of depression were ameliorated (p = 0.001) in the study group than control group
Jaggers et al. (2015) USA	Asymptomatic (63%), Symptomatic (10%), AIDS patients (25%), Missing report (2%) 36 Participants on ART 18 years & older Male (37), Female (12) NR (44) N = 93 52.69% (47.31%)	Supervised combined aerobic & resistance training for 50 min—(Aerobic exercise: 30 min on treadmill at 50–70% MHR; Resistance training: 20 min of 1 set, 12 repetitions), 2 sessions/week for 6 weeks n = 46(26) 2 missing data NR	6 weeks	Engaged in a sedentary lifestyle n = 47(23) 3 missing data	Depression	POMS-D	Symptoms of self-reported depression were ameliorated (p = 0.03)
Roos et al. (2014) South Africa	NR On HAART 20–65 years Male (18), Female (66) N = 84 (60.7%) 39.3%	Participants received a pedometer & activity diary that included education material & documents for self-monitoring Brisk walking was encouraged at 60–75% of the age-predicted maximum heart rate Participants received 5 monthly contact sessions & 1 cellphone SMS as motivation Incremental walking program started at 1000 steps/day from participants’ baseline step count, at 3 times/week Step count was adjusted with additional 500 steps every 2 weeks when participants attained their preceding goal until a value of 3000 steps from baseline was achieved After reaching the 3000-step count goal, frequency/week was adjusted from 3 to 4 to 5 times/week only if participants reached their previously determined frequency & managed well without physical complaints 42 (29) 72.4%	12 months	Control group continued with standard clinic Management & received 1 phone call monthly from the researcher to determine participants’ health status 42 (22)	Physical Activity Level	Yamax SW200 Pedometer	Physical activity level was not significantly improved but participants exceeded the optimum (3000 steps/day) public Health recommendation
Maharaj and Chetty (2011) South Africa	NR On HAART 18 & older Male (34), Female (18) N = 52 69% (31%)	Total duration of 40 min Supervised aerobic exercise: on a cycle ergometer & treadmill for 20 min each with a rest period of 20 min once a week for 12 weeks Home programme: 10 min of brisk walking, squatting & jogging 3 times/week for 12 weeks 26(20) 77%	12 weeks	Received 20 min of Heat therapy on the thigh muscles using shortwave diathermy plus a reading of magazines at home for 30 min, 3 times/week for 12 weeks 26(16)	Social functioning/Social participation	The social functioning domain of SF-36 MOS	Social functioning was improved (p = 0.022)
Ogalha et al. (2011) Brazil	NR On ARV drugs 18 & older Male(34), Female(29) (Gender of 7 dropouts in the control group was not reported) N = 70 90% (10%)	One hour supervised gym class plus monthly nutritional counselling 3 times/week for 24 weeks 35(35) 70%	24 weeks	One-hour monthly discussion on nutritional needs/recommendations & the importance of regular physical activity 35(28)	Social functioning/Social participation	The social functioning domain of SF-36 MOS	QOL was improved unlike social functioning
Tiozzo, (2011) USA	NR On HAART 18 years & older Male(14), Female(9) Gender of 14 dropouts from both control & exercise group was not reported N = 37 62.16%(37.84%)	Supervised Combined Aerobic & Resistance Exercise Training (CARET): 10-50 min of aerobic exercise at 60–75%MHR plus core exercises:3 sets of 15–20 repetitions at 60–75% 1RM & resistance exercises of 1 set 8–12 repetitions at 60–70% 1RM for 3 times/week for 12 weeks 12(6) 81%	12 weeks	No exercise participation. Telephoned every 4 weeks to maintain contact 11(8)	Social functioning/Social participation	The social functioning domain of SF-36 MOS	Physical & mental QoL improved relatively but not social functioning
Mutimura et al. (2008) Rwanda	NR On HAART 21–50 years Male(40), Female(60) N = 100 97% (3%)	15 min of brisk walking plus 45-60 min of supervised aerobic & strengthening exercises at 45–75% of MHR 3 times/week for 6 months The total duration of 1 h 30 min 50 (48) 82.2%	6 months	No exercise 50 (49)	Social relationship/social participation	The social domain of WHOQOL-BREF	self-esteem & social life improved (P < 0.001)
Neidig et al. (2003) USA	Asymptomatic, non-AIDS patients and Symptomatic, non-AIDS patients 75% On ART 18 years & above Male(52), Female (8) N = 60 80% (20%)	60 min of supervised aerobic exercise on either treadmill, cycle ergometer or walking at 60–80% VO_2_ Max, 3 times/week for 12 weeks 30(18) NR	12 weeks	Maintain usual activity n = 30(30)	Depression	POM-D CES-D BDI	Depressive symptoms was ameliorated as measured with the CES-D (p = 0.028) & POM-D scores (p = 0.045) but not on the BDI (p = 0.64)
Baigis et al. (2002) USA	Non-AIDS defining condition NR 24–61 years Male (79), Female (20) NR (24) N = 123 78.8% (21.2%)	Home-based Programme: 20 min workout on FM 340 Fitness Master Ski Machine at 75–85% MHR 3 times/week for 15 weeks 68(52) 71.1%	15 weeks	Usual care: 30 min visit/week for 15 weeks plus two phone calls/week 55(47)	Social functioning/social participation	MOS-HIV DASI	social functioning or participation did not improve

*NR* not recorded; *RM* repetition maximum; *MHR* maximum heart rate; *HRR* heart rate reserve; *VCT* Voluntary Counseling and Treatment Center; *GHQ-28* General Health Questionnaire; *HRQOL* Health Related Quality of Life; *MOS-HIV* Medical Outcome Study-HIV Health Survey; *CES-D* Center for Epidemiological Studies-Depression scale; *BDI* Beck’s Depression Inventory; *POM-D* Profile of Mood State-Depression subscale; *WHOQOL-HIV* World Health Organization Quality of Life HIV Health Survey; *DASI* Duke Activity Status Index; *SF-36 MOS* Short Form-Medical Outcome Study 36; *VO*_*2Max*_ maximum oxygen consumption

This review included 13 studies with 779 participants, with a gender distribution of 378 males and 310 females, while 91 participants (11.68%) did not state their gender (Table 1). Only 596 (76.51%) participants completed the study while 183 (23.49%) withdrew. The male/female ratio is approximately 1.2:1. Participants in the studies were 18 years of age or older. Ten studies included participants receiving antiretroviral therapy [43, 51, 53, 54, 56–60, 62]. Nevertheless, Neidig, Smith [53] and Jaggers, Hand [43] reported that only 75% of participants in their studies (35 out of 60) and 38.71% (36 out of 93), respectively, were using ART. However, three studies [52, 55, 61] did not report the ART status of the participants. Similarly, ten studies did not report the HIV staging of participants [43, 52, 54, 56–62]. On the other hand, two studies [51, 53] reported that according to the Centre for Disease Control's classification of HIV infection, the participants' disease was in categories A and B, i.e., in the asymptomatic and symptomatic stages, respectively. Additionally, one study [55], reported that all the participants were in the non-AIDS category (i.e., “category A"). However, one study [43] reported that the participants' HIV staging ranged from "asymptomatic" (63%), symptomatic (10%), AIDS (25%), and undisclosed (22%).

Further details on the participants' characteristics in this review are provided below:

(i) *Depression* The five publications [43, 51–54] that evaluated the effects of physical exercise on depression included 279 participants (Table 1). Upon completion of the study, 205 (73.48%) participants were retained, and 74 (26.52%) participants withdrew (Fig. 1). A total of 175 males, 60 females, and 44 participants with an undisclosed gender participated in the study. Men outnumber women in the review participants by a ratio of 3:1. No study reported on whether participants were taking any antidepressants. Among the studies, two were conducted in the USA [43, 53], while the remainder were conducted in developing countries including Nigeria [51], Iran [52], and Brazil [54].
(ii) *Physical activity level* There were two RCTs [61, 62], which investigated the effects of physical exercise training on PAL in PLWHA, involving 97 participants, aged 18–65. Participants included 26 males, 69 females, and 2 who did not disclose their gender. There is a male to female ratio of 1:2. On completion of the study, only 62 participants were retained while 35 participants withdrew. (Table 1).
(iii) *Social participation* Seven RCTs [54–60] were included for social participation and involved 447 participants, aged 18–86 years. They included 236 males, 166 females and 45 participants with an undisclosed gender. The male/female gender ratio is 1.4:1. On completion of the study, only 373 (83.45%) participants were retained while 79 (17.67%) participants withdrew. Two studies [54, 58] were located in Brazil, while two other studies [55, 60] were located in the USA. One study each was located in Hong Kong [59], South Africa [56], and Rwanda [57]. (Table 1)

### Outcome of intervention

#### Primary outcome

i. *Depression* The five included studies (Table 1) for depression assessed the participants using different measuring tools, namely: the profile of mood state questionnaire—POMS-30 [43, 53], General Health Questionnaire-28—GHQ-28 [52], Beck’s Depression Inventory-BDI [51, 53, 54], and Centre for Epidemiologic Studies Depression Scale (CES-D) [53].

#### Secondary outcomes

i. *Social participation* A total of seven studies (Table 1) included in our review for social participation/functioning used different instruments to measure participation/functioning, including the 36-Item Short Form Survey (SF-36) [56, 58–60], The Medical Outcomes Study HIV Health Survey (MOS-HIV) [55], Duke Activity Status Index [DASI]) [55], and The World Health Organization's Quality of Life HIV instrument-Brief (WHOQOL-HIV-BREF) [54, 57].
ii. *Physical activity level* The two studies on PAL (Table 1) used different measuring tools. Actigraph GT3X + Tri Axis Accelerometer was used in one study [61] to measure physical activity, while Yamax SW200 Pedometer measured the same variable in another study .

### Quality appraisal and risk of bias assessment

The risk of bias within the included studies is provided in Table 2. Performance bias (the lack of subject and therapist blinding) was the major source of bias in all the studies included. Based on the PEDro scale, ten studies (76.92%) were considered fair/moderate quality [43, 51–53, 55, 57–61]. Two studies (15.38%) were rated as high quality studies [56, 62] and one study (7.69%) was rated as low-quality [54]. Further details are provided below:

i. *Eligibility criteria* The authors from thirteen [15] of the included studies described their recruitment and screening criteria for their respective studies. Hence, the studies were considered as having low risk of bias in this regard.
ii. *Random allocation* The thirteen studies applied the randomisation procedure in allocating the eligible participants to the different arms of the study. Thus, they were considered as free of selective reporting bias.
iii. *Concealment of allocation* Concealed allocation was not done in eleven (84.61%) studies [43, 51–54, 56–61], detection bias for not reporting or providing enough information about blinding of the assessor in eleven (84.61%) studies [43, 51–55, 57–61] and no Intention to treat analysis in ten (76.92%) studies [ 43, 51–54, 57–61].
iv. *Baseline comparability* Baseline comparability was demonstrated across the groups in all the included studies except in two (15.38%) studies by Chung et al. [59] and Oliveira et al. [54]. The study groups in the two studies [54, 59] were non-equivalent at baseline and were considered as having a high risk of bias in this regard.
v. *Bias on blinding* The assessor and personnel blinding were reported in two (15.38%) studies [56, 62] and for which they were considered as having a low risk of bias in this regard.
vi. *The bias of outcome measurement from* < *85% of initial participants (incomplete outcome data)* Four (30.77%) studies reported adequate follow-up [57–59, 61] (Table 1). Overall, 183 out of 779 participants at baseline withdrew from the included studies accounting for 23.49% of the total number of participants. Withdrawal rates within individual studies ranged from 4.76% [59] to 60.7% [62] (Table 1). However, a moderate risk of attrition bias exists as eight [37, 43, 51, 52, 54–56, 60, 62] of the thirteen included studies (61.54%) reported withdrawal rates of > 15%. Three participants withdrew from the trials in one study [57]. The retention rate in three (23.08%) studies [57–59] was 90% to 97% due to low attrition, and therefore considered as having a low risk of incomplete outcome bias. The comparison groups in the included studies showed similar withdrawal rate. There  were participant(s) who did not comply with their exercise intervention or withdrew from the trials in nearly all the included studies. The adherence rate to the prescribed exercises ranged from 60% [61] to 96.3% across eight studies [54–61].

**Table 2:** A Quality appraisal using the PEDro scale

Study	Random allocation	Concealed allocation	Baseline comparability	Blinding of subjects	Blinding of Therapists	Blinding of assessor	Adequate follow-up	Intention to treat analysis	Between-group comparison	Point estimates and variability	Total score	Quality index
Chung et al. (2019)	Yes	No	No	No	No	No	Yes	No	Yes	Yes	4/10	Moderate
Oliveira et al. (2019)	Yes	No	No	No	No	No	No	No	Yes	Yes	3/10	Low
Dianastinab et al. (2018)	Yes	No	Yes	No	No	No	No	No	Yes	Yes	4/10	Moderate
McDemortt et al. (2016)	Yes	No	Yes	No	No	No	Yes	No	Yes	Yes	5/10	Moderate
Aweto et al. (2016)	Yes	No	Yes	No	No	No	No	No	Yes	Yes	4/10	Moderate
Jaggers et al. (2015)	Yes	No	Yes	No	No	No	No	No	Yes	Yes	4/10	Moderate
Roos et al. (2014)	Yes	Yes	Yes	No	No	Yes	No	Yes	Yes	Yes	7/10	High
Ogalha et al. (2011)	Yes	No	Yes	No	No	No	Yes	No	Yes	Yes	5/10	Moderate
Maharaj et al. (2011)	Yes	No	Yes	No	No	Yes	No	Yes	Yes	Yes	6/10	High
Tiozzo (2011)	Yes	No	Yes	No	No	No	No	No	Yes	Yes	4/10	Moderate
Mutimura et al. (2008)	Yes	No	Yes	No	No	No	Yes	No	Yes	Yes	5/10	Moderate
Baigis et al. (2002)	Yes	Yes	Yes	No	No	No	No	Yes	No	Yes	5/10	Moderate
Nedig et al. (2003)	Yes	No	Yes	No	No	No	No	No	Yes	Yes	4/10	Moderate

### Meta-analyses: Effects of interventions

This review carried out three meta-analyses for the included studies on depression, PAL and social participation.

#### Depression

Four [43, 51, 52, 54] (i.e. 80%) of the five studies [43, 51–54] included in this review, reported that physical exercise training significantly reduced the symptoms of depression in PLWHA (Table 3). However, none of the studies provided information on whether the participants were on antidepressant medications or not. The prescriptions of physical exercise training that ameliorated the symptoms of depression in the four studies were:

i. Combined exercise training (aerobic exercise: 40–45% Maximum Heart Rate for 45 min) plus (Strength training exercise: 3 sets of 8 repetitions on 50–55% Repetitive Maximum for 15 min); 3 × per week [52]
ii. Aerobic exercise training using a cycle ergometer at 50–60% Heart Rate Reserve for 40 min per session; 3 × per week for 6 weeks [51]
iii. Combined exercise training: aerobic exercise (30 min on treadmill at 50–70% Maximum Heart Rate) and resistance exercise (upper and lower-body resistance training: 1 set of 12 repetitions each on plate-loaded Hammer Strength Machines; upper anterior and posterior legs on Life Circuit Machines; free weights), 50 min/session; 2 ×/week for 6 weeks [43] and
iv. Combined exercise training (CET): aerobic exercise (15–20 min of moderate-intensity at 50–65% HRR), and strength training (15–20 min of 8–15 MHR of 2–3 sets), 3 sessions per week for 16 weeks [54].

**Table 3:** Outcome values for depression

Study	Timepoint—immediately post-intervention	Depression (measurement tool)
Oliveira et al. (2019)	✓	Minimal depression—{Int. *(5 (55.6) vs Cont. *(13 (92.9)); p < 0.05; d = 0.01} (BDI) Mild, Moderate or Severe depression—{Int. (1 (7.1)) vs Cont. (4 (44.4)); p < 0.005; d = 0.17} (BDI)
Dianastinab et al. (2018)	✓	{Int. (2.69 ± 1.44) vs Cont. (7.60 ± 5.38); p = 0.008; d = NR} (GHQ-28-Severe Depression)
Aweto et al. (2016)	✓	{Int. (3.50 ± 1.27) vs Cont. (8.33 ± 5.80); p = 0.001; d = NR} (BDI)
Jaggers et al. (2015)	✓	{Int. (6.21 ± 1.50 ͣ) vs Cont. (8.2 ± 2.08 ͣ); p < 0.05; d = NR} (POM-D)
Nedig et al. (2003)	✓	{Int. (6.1 ± 8.9) vs Cont. (10.9 ± 11.2); p = 0.045; d = NR} (POM-D) {Int. (7.2 ± 7.1) vs Cont. (14.1 ± 11.3); p = 0.028; d = NR} (CES-D) {Int. (5.6 ± 6.3) vs Cont. (8.7 ± 7.1); p = NS; d = NR} (BDI)

*Int* Intervention group; *Cont* Control group; *p* p-value; *d* effect size; Except otherwise stated, outcomes are reported as: [Int (Mean ± SD) vs Cont (Mean ± SD); p-value; d (effect size)]

BDI = Becks Depression Inventory; GHQ-28-Severe Depression = General Health Questionnaire-Severe Depression Sub-scale score; POM-D = Profile of Mood Scale-depression score; CES-D = Center for Epidemiological Studies-Depression scale

ͣReported as Standard Error (SE)

*Values are reported as [Int. absolute(relative) scores vs Cont. absolute(relative) scores; p-value; d (effect size)]

**Fig. 2:**
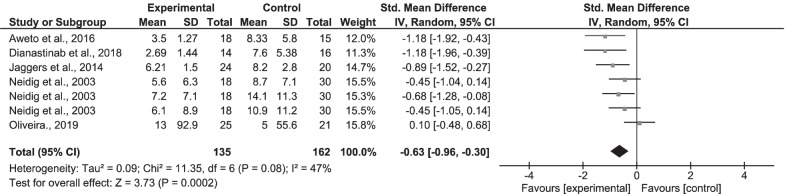
Forest plot for depression—meta-analysis of five studies

For the post-intervention analysis, we found a moderate standardized mean difference (SMD = − 0.63, 95% CI − 0.96, − 0.30) in favour of the physical exercise group in the random-effect model, for all the five studies included for depression [43, 51–54]. Thus, physical exercise training had an overall significant positive effect (Z = 3.73, p ≤ 0.0002; 5 studies; 205 participants) on symptoms of depression compared to the control group (Fig. 2). A decrease in depressive symptoms was a significant trend for participants in the physical exercise compared to the no-exercise group; aerobic exercise compared to normal routine activity group; aerobic and resistance exercise compared to other control groups. The included studies used several measuring tools namely: GHQ-28; BDI; POMS-D; CES-D. (Table 1).

#### Physical activity level

**Fig. 3:**
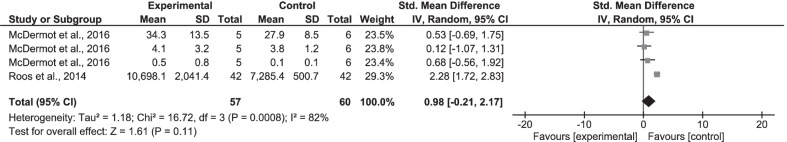
Forest plot for physical activity level—meta-analysis of two studies

One [62] of the two [61, 62] studies included in this review showed that exercises significantly improved the PAL in PLWHA between 0—6 months but not between 6 and 12 months (Table 4). For both studies, a large standardized mean difference (SMD = 0.98, 95% CI − 0.25, 2.17) in favour of the control group was found in the random-effect model for post-intervention values. Thus, physical exercise training had no overall significant effect (Z = 1.61, p = 0.11; 2 studies; 62 participants) on PAL in the experimental group compared to the control group. (Fig. 3). Nevertheless, the overall trend was that the experimental groups consistently recorded increased post-intervention PAL compared to the baseline values and vice versa in the control groups. The between-group comparison also showed that the post-intervention PAL was increased mainly in the experimental groups compared to the controls across the studies. The measuring tools used from the included studies were: Actigraph GT3X + Tri Axis Accelerometer; Yamax SW200 Pedometer. (Table 1).

**Table 4:** Outcome values for PA level Post Intervention

Study	Timepoint–Immediately post Intervention	Physical activity level (Unit of measurement)
McDermott et al. (2016)	✓	LPA = {Int (34.3 ± 13.5) vs Cont. (27.9 ± 8.5); p = NS; d = NR} (Accelerometer hours/week) MPA = {Int (4.1 ± 3.2) vs Cont. (3.8 ± 1.2); p = NS; d = NR} (Accelerometer hours/week) VPA = {Int (0.5 ± 0.8) vs Cont. (0.1 ± 0.1); p = NS; d = NR} (Accelerometer hours/week)
Roos et al. (2014)	✓	{Int (10,698.1 ± 2041.4*) vs Cont. (7285.4 ± 500.7*); p = 0.49; d = NR} (Pedometer Step count/day)

Int = Intervention group; Cont. = Control group; p = p-value; d = effect size; LPA = Light Physical Activity; MPA = Moderate Physical Activity, VPA = Vigorous Physical Activity

Except otherwise stated, outcomes are reported as:

[Int (Mean ± SD) vs Cont. (Mean ± SD); p-value; d (effect size)]

*Outcome reported as Standard Error

#### Social participation

A significant improvement in social participation due to physical exercise training (Table 5) in PLWHA was reported in three (42.86%) [56, 57, 59] out of the seven included studies. In contrast, a significant decrease in social participation due to physical exercise training was reported in one paper [58]. The prescriptions of physical exercise training that improved social participation in the three studies were:

i. Supervised combined aerobic and resistance physical exercise training, respectively, at a moderate intensity of 50–70% MHR, for 45 min per session, 2 sessions per week, for 8 weeks [59]
ii. Brisk walking for 15 min plus supervised aerobic and strengthening exercises at 45–75% of MHR for 45-60 min per session, (a total exercise duration of 1 h 30 min), 3 times per week, for 6 months [57]
iii. Supervised aerobic exercise on a cycle ergometer and treadmill for 20 min each with a rest period of 20 min once a week for 12 weeks, plus home programme: 10 min of brisk walking, squatting and jogging 3 times per week for 12 weeks [56]

**Table 5:** Outcome values for Social Participation

Study	Timepoint’-Immediately post Intervention	Social functioning/social participation (measurement tool—domain)
Chung and Lou (2019)	✓	{Int (97.50 ± 5.27) vs cont. (81.25 ± 16.93); p = 0.043; d = NR} (SF-36—Social domain)
Oliveira et al. (2019)	✓	{Int (15.0 ± 2.4) vs cont. (13.9 ± 2.9); p = NS; d = 0.07} (WHOQOL-HIV—Social domain)
Maharaj et al. (2011)	✓	{Int (70.2 ± 18.9) vs cont. (66.8 ± 14.4); p = 0.022; d = NR} (SF-36—Social domain)
Ogalha et al. (2011)	✓	{Int (91.8 ± 31.6) vs cont. (94.0 ± 10.9); p = 0.001; d = NR} (SF-36—Social domain)
Tiozzo (2011)	✓	{Int (81.3 ± 20.9) vs cont. (86.1 ± 26.1); p = NS; d = NR} (SF-36—Social domain)
Mutimura et al. (2008)	✓	{Int (9.8 ± 0.7) vs cont. (9.0 ± 0.5); p < 0.001; d = NR} (WHOQOL-HIV- Social domain)
Baigis et al. (2002)	✓	{Int (88.4 ± 22.1) vs Cont. (84.9 ± 21.2); p = NS; d = 0.57} (MOS-HIV—social domain)

Int = Intervention group; Cont = Control group; p = p-value; d = effect size; Except otherwise stated, outcomes are reported as: [Int (Mean ± SD) vs Cont (Mean ± SD); p-value; d (effect size)]

SF-36 = Short Form 36; WHOQOL-HIV = World Health Organisation Quality of Life HIV Health Survey; MOS-HIV = Medical Outcomes Study-HIV Health Survey

The only exercise training prescription that showed significantly lower values for social participation than control was:

i. Supervised gym class, for 1 h, 3 times per week for 24 weeks plus monthly nutritional counselling [58]. However, the baseline data indicated that the intervention group also had a significantly lower mean value for social participation compared to the control.

**Fig. 4:**
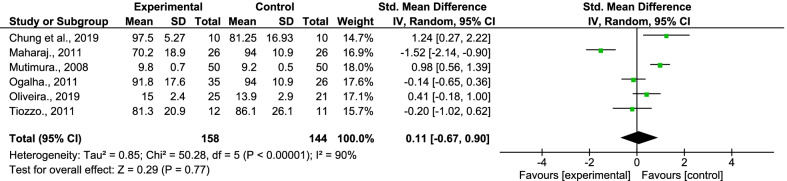
Forest plot for social participation—meta-analysis of seven studies

Data from one paper by Baigis et al. [55] was not pooled for the meta-analysis because the standard deviation values were not provided by the journal editorial team (as the authors’ contacts/email addresses were not provided in the publication) even after several correspondences. For the six studies [54, 56–60] pooled for meta-analysis for social participation, a small standardized mean difference (SMD = 0.11, 95% CI − 0.67, 0.90) was reported in favour of the control group in the random-effect model. Therefore, physical exercise training had no significant effect on social participation (Z =0.29 , p = 0.77; 6 studies; 368 participants) (Fig. 4) among participants: in the exercise-group compared to no exercise group; aerobic exercise compared to normal routine activity group; aerobic gym class plus nutritional counselling to discussion on nutritional needs, and resistance exercise compared to other control groups. Measuring tools used in the included studies were: SF-36 MOS, WHOQOL-HIV-BREF, WHOQOL-HIV, MOS HIV DASI. (Table 1).

#### Heterogeneity

The data from the primary meta-analysis for depression showed a moderate/medium heterogeneity (I^2^ = 47%, X^2^ = 11.35, df = 6, p = 0.08) (Fig. 2—forest plot). In contrast, the data for the primary meta-analysis for PAL showed substantial/high heterogeneity (I^2^ = 82%; X^2^ = 1.61, df = 3, p < 0.0008) (Fig. 3) and likewise for social participation (I^2^ = 90%, X^2^ = 50.28, df = 5, p < 0.00001) (Fig. 4).

### Sensitivity analysis

#### Depression

**Fig. 5:**
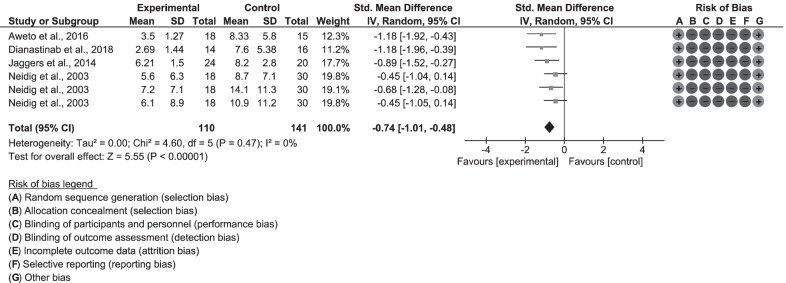
Sensitivity analysis for depression—secondary meta-analysis of four studies

After the primary meta-analysis, the first sensitivity analysis was done for depression, and which excluded the trials by Oliveira et al. [54] because the control and exercise groups were non-equivalent at baseline. A significant effect was found (Fig. 5) for exercise intervention (SMD = 0.74 [95% CI − 1.01, − 0.48], Z = 5.55; p = 0.00001), and the statistical heterogeneity was low (I^2^ = 0%, X^2^ = 4.60, df = 5, p = 0.47).

#### Social participation

**Fig. 6:**
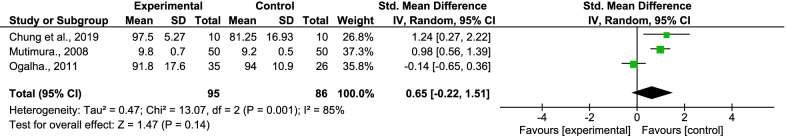
Sensitivity analysis for social participation—secondary meta-analysis of four studies

Subsequent to the primary meta-analysis (Fig. 6), the sensitivity analysis done for social participation excluded three clinical trials in which the attrition rate was greater than 15% [54, 56, 60], and found no significant effect for physical exercise training (SMD = 0.65 [95% CI − 0.22, 1.51], Z = 1.47; p = 0.14). However, the statistical heterogeneity was high (I^2^ = 85%, X^2^ = 13.07, df = 2, p = 0.001).

## Discussion

### Depression

The combined evidence from the included studies seems to support the potential of physical exercise training to ameliorate the symptoms of depression in PLWHA. The synthesised evidence in this review revealed that aerobic exercises or combined (aerobic + resistance) exercises performed 2–3 times/week, at 40–60 min per session, and for between 6 and 24 weeks, effectively ameliorated the symptoms of depression in PLWHA. However, there were three times lesser females than males in the studies included in this review for depression. This might have some implications as a previous epidemiological study [63] in PLWHA revealed that symptoms of depression were more prevalent in women than men. The effect size in the meta-analyses was large, and therefore both males and females would have benefitted from the anti-depressant- effect of physical exercise training.

Additionally, as the upper limit of age in the individual studies was not above 65 years, it is expected that age will not contribute significantly to the distribution of depressive symptoms among the participants. For depression, the sensitivity analysis excluded one study [54] for the non-equivalence of the control and exercise groups at baseline, and which resolved the medium heterogeneity of the primary meta-analysis. This suggests that some flaws exist in the randomisation process of the included studies, and need to be addressed in future studies. As the excluded study [54] was conducted in a developing country (Brazil), it is reasonable to assume that one of the challenges in conducting RCTs in resource-limited settings may be related to the availability of the technology for implementing randomisation, which may vary from the developed clime.

The findings of this study agree with the evidence from other systematic reviews [1, 3, 64] that physical exercise training ameliorates the symptoms of depression in PLWHA. Besides, several systematic reviews [42, 65, 67] present evidence that aerobic and resistance exercise intervention at various intensities have both stand-alone and combined positive effects on various aspects of mental health or psychological outcomes, such as depression [40, 66–68], hope, desire to continue living [65], and health-related quality of life [65] in PLWHA. O’Brien et al. [3] presented evidence that performing an aerobic exercise or a combination of aerobic and resistance exercises, three times per week, for at least five weeks, improved the symptoms of depression in adults with HIV.

A recent systematic review among PLWHA by Heissel et al. [64] also revealed that physical exercise has a large effect on depression (p = 0.02) compared to controls. However, the review included nine studies on both conventional and unconventional exercises like yoga, which is considered a mind–body practice that integrates various body poses, breathing techniques, and utilises deep meditation [69]. Invariably, the synthesised evidence by Heissel et al. [64] cannot be strictly attributed to the effects of physical exercises alone, unlike our study which included only studies that investigated conventional exercises alone. The presence of confounding factors may explain why the results from Heissel et al. have a higher statistical heterogeneity (I^2^ = 94%; p = 0.02) and weakens our confidence in the estimate of effect. In contrast, our study has a lower heterogeneity and highlights the positive effect of exercise on symptoms of depression in a more homogenous sample of PLWHA. A lower heterogeneity increases our confidence in the estimate of effects of physical exercise training on symptoms of depression, and point to a significant role for physical exercise in the management of depression in PLWHA.

We further explored the literature for theoretical projections on the molecular and morphological basis for the antidepressant-like effect of exercise training on vital brain targets. The literature revealed that aerobic exercises could increase the growth or size (through neurogenesis) and function of the hippocampus thereby helping the brain to exert control actions that minimise oxidative stress, attenuate inflammation [70] and ameliorate depression [71]. In essence, exercise creates differing effects to the actions of the facilitators of depression such as diminished neurogenesis, which in depressed individuals indicates hippocampal volumetric decreases [72], and predisposition to experiencing a depression episode [72]. Running on a treadmill also helps alleviate depression by stimulating the wingless (Wnt)-frizzled (Fz) signalling pathways which is an upstream regulator/inhibitor of glycogen synthase kinase 3 (GSK 3) that is linked to depression [73]. A significant increase in GSK-3 expression impairs hippocampal neurogenesis and increases depression risk [74]. Exercise, especially resistance exercise, promotes the level of serotonin in the brain of humans, which facilitates cell proliferation and neuroplastic changes associated with antidepressant effects [75]. As well, exercise increases synaptic plasticity in hippocampus-prefrontal cortex neuronal pathways [71], which are typically compromised by depression [76]. Thus, in order to improve how depression is managed in PLWHA, the significance of exercise in promoting synaptic plasticity (when antidepressant treatments do not increase cell proliferation) and in maintaining brain function by stimulating the Wnt signalling channel, should be explored.

### Physical activity level

As far as we know, this is the first systematic review to demonstrate the effects of physical exercise on PAL in PLWHA patients. Though the meta-analysis from the two studies [61, 62] included for PAL showed no evidence that exercise training improves the PAL in PLWHA, however, the findings of both studies revealed a consistent trend of improved post-intervention PAL (though not significant) among the experimental groups compared to the controls. Since the overall effect was not significant, it implies that physical exercise training may not improve the PAL among PLWHA compared to no exercise group; aerobic exercise compared to normal routine activity group; aerobic and resistance exercise compared to other control groups. Nevertheless, there are only a few studies done in this area making it difficult to form a scientific opinion on this matter, especially as the two studies [61, 62] included in this review revealed a mixed result and therefore, lack of scientific consensus in this area.

McDermott et al. [61] reported no significant difference (p > 0.05) in the PAL between the experimental and control groups after 16 weeks of exercise intervention. However, the study did not show any deterioration in the PAL as well. This implies that exercise intervention should have a protective effect in maintaining the PAL even if it does not significantly boost it. However, the results of the study should be interpreted with caution, because the sample size was small (n = 13) and the number of participants that completed the study was even smaller (n = 11). In essence, the study was underpowered to detect the statistical difference between the two groups. Invariably, there is a possibility that the true effects of exercise training on PAL in PLWHA were underestimated in the study due to type II error. More so, there is no report regarding the effect size to estimate the clinical significance of their findings. On the other hand, Roos et al. [62] demonstrated that physical exercises significantly boosted the PAL of PLWHA within six months, but not beyond 6–12 months, whereby the compliance of the participants was lower than the first 6 months. Consequently, the findings demonstrate that unsupervised pedometer-based physical activity intervention can translate into tangible health benefits. This approach can be scaled-up to population-level to enable more active lifestyles for PLWHA if compliance is high as was the case from 0 to 6 months. Nonetheless, it is not certain to what extent an unsupervised physical activity behavioural intervention approach will translate to a sustainable behavioural/lifestyle change considering that the participants recorded a low compliance level between 6 and 12 months. It probably explains why the post-intervention assessment of PAL showed no significant difference between the intervention and control groups.

The above findings highlight the need to structure the physical activity review appointments, behavioural evaluations and goal-setting sessions within a common theoretical framework when implementing any walking strategy to tackle the barriers to physical activity [77]. Such a theoretical framework aids the individuals to find barriers to walking, and make choices on how to alter and improve their walking behaviour [78] using various strategies. McDermott et al. [61], and Roos et al. [62] did not apply this approach and seemed not to have implemented regular physical activity consultation sessions in their studies. This may partly explain why their studies were unable to find a significant difference in the PAL when the intervention group was compared to the control group. Notwithstanding this gap, the higher post-intervention PAL (though not significant) compared to the baseline value buttresses that physical exercise training boosts PAL in PLWHA. It reaffirms the evidence from McDermott et al. [61] that physical exercise training may have a protective effect in preventing a decline in the PAL of PLWHA even if it does not significantly increase the PAL. Importantly, the quality of the study by Roos et al. [62] is high and the sample size (n = 84) is sufficiently powered to detect the difference between the groups but was affected by the high attrition rate (> 15%), or else, the findings would have been more significant. Overall, the paucity of literature, apart from the observed flaws in the two studies highlighted above, warrants the conduct of more high-quality RCTs to determine the true estimate of the effect of exercises on PAL in PLWHA, which is important to guide practice.

### Social participation

The available evidence demonstrates that the overall effect of exercise on social participation in PLWHA is not significant. However, we found a high attrition rate (> 15%) in all the included studies except three [57–59], which may influence the outcome of the findings. Besides, the heterogeneity of the primary meta-analysis was high, and likewise the sensitivity analysis. Therefore, the data from the included studies do not seem to represent the true estimate of effect in a homogenous population of PLWHA. The heterogeneity of the data may be related to the diverse measuring tools for social participation/functioning employed across the included studies. Also, there is a selection bias as five [54, 56–59] of the seven papers did not implement the concealed allocation of the participants into the study groups. One of the studies with selection bias [58] reported a significant post-intervention decline in social participation in the intervention group than the control. However, the true estimate of effect is in doubt because the baseline value of social participation was significantly higher in the control group than in the intervention group probably due to flaws in the randomisation procedure. Despite this limitation, exercise training did not reduce social participation below its baseline value in the intervention group. In essence, social participation neither deteriorated nor significantly improved among the exercising participants compared to control. Also, there is a likelihood that age might have some effects considering that older adults, up to 86 years of age, were involved in the included studies. Hence, the findings of our study do not agree with the results of previous studies in non-HIV infected population including a social experiment [79], a systematic review [80], a descriptive cross-sectional study of individuals with disabilities [82], and an RCT of stroke survivours [81]. Similarly, our study presented evidence at variance with the estimate of effect reported by another systematic review by Vancampfort et al. [83] which included only studies involving PLWHA but was not restricted to RCTs.

All the cited studies above [79–83] identified a link between physical activity profile and social participation probably due to the effects of physical activity on mental health, especially mood [84–86]. However, several limitations related to study design, and the involvement of mostly non-HIV infected population do not allow for the comparisons of the results between our study and the above-cited studies. Nevertheless, the importance of our study is supported by a previous view that HIV and rehabilitation research should focus on social participation [87]. In an HIV population, it is especially important as social stigmatization is problematic for PLWHA in different countries of the world [88]. Social stigmatisation in PLWHA may lead to low self-esteem, mood disorders, and withdrawal from social activities or social isolation [88] resulting in a sedentary lifestyle or a decline in ambulatory function. Meanwhile, the ambulatory function is often restricted with polypharmacy, and a subsequent rise in complications associated with HIV infection [86] may add to the restrictions or lack of participation in physical (sedentary lifestyle) and social activities. The resulting multi-system (neurological, musculoskeletal, cardiopulmonary and metabolic) dysfunction may impair walking function, as well as compromise the quality of life [26]. Limited social participation may also be related to a feeling of low self-esteem or rejection [89]. Therefore, any intervention that may improve mental health by improving mood, and self-esteem in PLWHA, may also improve their physical functioning and likewise social participation. Knapen et al. [90] analysed four meta-analyses on the effects of physical exercise on mental and physical health in people with mental health problems. They found that exercise training resulted in a better quality of life, reduced emotional stress, enhanced self-esteem, and body image which have implications for a social relationship, and may have translational benefit for PLWHA. It emphasizes the point earlier made by Dianatinasab et al. [91] that “keeping up physical activities and exercises are key strategies in medical and social care of PLWHA.”

### Limitations

There are several limitations to this study. First, the variables of interest are constructs, which are somewhat subjective and therefore difficult to measure objectively or accurately. Besides, the self-reported psychometric instruments used in data collection for the included studies are prone to recall bias. Importantly, the small sample size in some of the included studies will likely introduce bias for type II error, and likewise, the small number of studies that were included in this review that met the eligibility criteria. The application of the standard mean difference provided a comparative basis to evaluate the effects of physical exercise training on the outcomes across the studies, where diverse measuring tools were used. However, there are difficulties applicable to the interpretation of the SMDs, which may limit the true estimation of the effects of the intervention across the studies. For instance, the interpretation of the SMDs varies across instruments depending on whether improvement in a given instrument is associated with lower or higher scores on the outcome measure [53]. This is quite tricky when the SMDs have overlapping confidence intervals, which increases the failure to reject the null hypothesis. This also means an increase in type II error than the corresponding hypothesis test. Consequently, there is a need for more robust, rigorous and high-quality studies that will measure each variable of interest using more than one tool to enable realistic comparison of data across studies.

## Conclusion

The evidence from this study suggests that physical exercise training has a potential benefit in ameliorating the symptoms of depression in PLWHA. The antidepressant-like effects of physical exercise modalities fit into the major treatment goals of PLWHA, especially the need to improve PAL [which may be impeded by depression [92], recover strength and physical fitness [93] for productive social participation/functioning in a community. Therefore, the findings of this study recommend the integration of physical exercises into the routine care of PLWHA as a self-management strategy in the rehabilitation intervention to address the disability and mental health needs of PLWHA. This also reiterates the recommendation of previous authors [81]. Furthermore, the trend across the studies indicated that physical exercise training may prevent a decline but not significantly improve PAL and social participation in PLWHA.

### Implications for practice

Evidence regarding the impact of exercise training on depression favours exercise when compared to control and suggests that the symptoms of depression are ameliorated by exercise training. These findings have important implications for practice, especially in sub-Saharan African where despite the existing evidence of the beneficial value of physical exercises/activity in improving mental health, yet physical exercises/activity is neglected as a routine rehabilitation modality in the mental healthcare systems [83]. Importantly, mood disorders, which is common in PLWHA, have also been linked to poor adherence to medications and consequently, poor health outcomes [1, 2, 36–39]. If physical activity/exercises ameliorate the symptoms of depression, it may also improve adherence to medication and likewise other related health outcomes. Therefore, public health policies and initiatives designed to increase participation in exercises may have the potential to improve mental health and general wellbeing among PLWHA.

### Implications for research

Across the included studies, there were notable potential sources of bias related to baseline comparability, incomplete outcome reporting, small sample size, short intervention duration, non-implementation of blinding of therapists and assessors. Also, diverse outcome measuring tools with different validity and reliability values were used in the included studies, which made data comparison across the studies more difficult. Some studies that evaluated the effects of exercises on depression and PAL were underpowered to detect the differences in the mean between the intervention and control groups. Some of the studies did not provide information on the adherence rate and effect size to enable the estimation of the effect of the physical exercise training on the variables of interest in the PLWHA. Similarly, information on whether the participants were on antidepressant medications before the study was not provided in any of the studies. This makes it difficult to determine whether the antidepressant-like effects of physical exercises were independent or amplified in those taking antidepressant medications. These weaknesses may affect the validity of the findings of the various studies included in this review. Meanwhile, the paucity of RCTs on the impact of physical exercise training on PAL made it difficult to form a scientific opinion on its’ effect.

The variability in the number of participants across the studies was high for social participation, ranging from 21 participants in one study [59] to 100 participants in another study [57]. The same applied to studies on physical activity level which included participants ranging from 11 participants in one study [61] to 84 participants in another study [62]. In contrast, the studies on depression had the least variability of participants, ranging from 40 participants in one study [52] to 93 participants in another study [43]. This may contribute to the heterogeneity of the data across the studies, and might explain the substantial heterogeneity in the studies related to social participation and PAL. Additionally, the substantial heterogeneity suggests that there are some clinical or methodological differences, or both, across the studies included in the review. The is buttressed by the fact that the included studies showed variances in the study characteristics such as gender, location of study (developed versus developing countries), the type and dose of physical exercise training, measuring tools, sample size as well as non-equivalence of the study groups at baseline, and high attrition rates. Therefore, the variances also add to the clinical or methodological differences and suggest some flaws in the trials design, documentation and implementation which should be addressed in future studies.

## Supplementary Information

**Additional file 1: Search strategy in PubMed for depression, physical activity level, and social participation.** Description of data: The MESH terms used to search the Pubmed database. For evidence of the effects of exercise training on depression, physical activity level and social participation in HIV conditions.

## Data Availability

The datasets used and/or analysed during the current study are available from the corresponding author on reasonable request.

## Abbreviations

HIV: Human immune deficiency virus
AIDS: Acquired immune deficiency syndrome
ART: Antiretroviral therapy
HAART: Highly antiretroviral therapy
PLWHA: People living with HIV/AIDS
ROB: Risk of bias
RevMan: Review Manager
RCTS: Randomized control trials
MeSH: Medical subject heading
AMED: Allied and Complementary Medicine Database
CINAHL: Cumulative Index to Nursing and Allied Health Literature
EMBASE: Excerpta Medica database
AMED: Allied and Complementary Medicine Database
PEDro: Physiotherapy Evidence Database
GRADE: Grading of Recommendations Assessment Development and Evaluation
INPLASY: International platform of registered systematic review and meta-analysis protocols
PRISMA: Preferred Reporting Items for Systematic Reviews and Meta-analyses
SMD: Standardised mean difference
MOS: Medical Outcomes Study
NHREC: Nigeria Health Research Ethics Committee
WHO: World Health Organisation
POMS-30: Profile of Mood State Questionnaire-30
GHQ-28: General Health Questionnaire-28
BDI: Beck’s Depression Inventory
MOCA: Montreal cognitive assessment
SDS: Symptom Distress Scale
PSS: Perceived Stress Scale
GS-ES: Generalized Self-Efficacy Scale
CES-D: Centre for Epidemiologic Studies Depression Scale
SF-36: 36-Item Short Form Survey
MOS-HIV: The Medical Outcomes Study HIV Health Survey
DASI: Duke Activity Status Index
WHOQOL-HIV-BREF: The World Health Organization's Quality of Life HIV instrument-Brief
