# Supplementary material for: Impact of exercise training on symptoms of depression, physical activity level and social participation in people living with HIV/AIDS: a systematic review and meta-analysis
Source: BMC Infect Dis. 2022 May 16;22:469. doi: 10.1186/s12879-022-07145-4 (PMC9109396; doi:10.1186/s12879-022-07145-4)
Supplement: Supplementary file 1 — Additional file 1: Search strategy in PubMed for depression, physical activity level, and social participation. Description of data: The MESH terms used to search the Pubmed database. For evidence of the effects of exercise training on depression, physical activity level and social participation in HIV conditions. [file 12879_2022_7145_MOESM1_ESM.docx]

**APPENDIX 1. SEARCH STRATEGY**

| **CONCEPT** | **SEARCH TERMS** |
| --- | --- |
| **Population** | 1. HIV patients 2. HIV/AIDS patients 3. HIV positive patients 4. People living with HIV/AIDS 5. People living with HIV OR HIV infections 6. Acquired immune deficiency syndrome 7. HIV/AIDS 8. HIV 9. AIDS 10. HIV seropositive patients 11. PLWH 12. PLWHA 13. HIV-1 NOT HIV-2 14. 1 OR 2 OR 3 OR 4 OR 5 OR 6 OR 7 OR 8 OR 9 OR 10 OR 11 OR 12 OR 13 |
| **Intervention** | 1. exercise intervention 2. Physical exercise 3. Exercise training 4. Aerobic exercise 5. Progressive resistance exercise 6. Strength training exercise 7. Resistance exercise 8. Exercise 9. Home based exercise 10. Supervised exercise 11. 15 OR 16 OR 17 OR 18 OR 19 OR 20 OR 21 OR 22 OR 23 OR 24 |
| **Study design** | 1. Randomised controlled trial 2. Clinical trials 3. Random allocation 4. Control groups 5. 26 OR 27 OR 28 OR 29 |
| **Outcome** | 1. mental health 2. Mental hygiene 3. Mental disorder 4. Social stigma 5. Social values 6. Psychology 7. Psychological status 8. Behaviour disorder 9. Psycho-social 10. Physical activity level 11. Accelerometry 12. Actigraph 13. Motor activity 14. Physical fitness 15. Activities of daily living 16. ADL 17. Sedentary behaviour 18. Social participation 19. Social determinants of health 20. Social behaviour 21. Social activities 22. Social isolation 23. Leisure activities 24. 31 OR 32 OR 33 OR 34 OR 35 OR 36 OR 37 OR 38 OR 39 OR 40 OR 41 OR 42 OR 43 OR 44 OR 45 OR 50 OR 51 OR 52 OR 53 25. 14 AND 25 AND 30 AND 54 |
